# The Influence of Circadian Rhythm on the Antioxidant Capacity of Saliva in Periodontal Diseases

**DOI:** 10.7759/cureus.56174

**Published:** 2024-03-14

**Authors:** Randa Diab, Antoine Choufani, Jihad Dagher, Nathalie Chahine

**Affiliations:** 1 Faculty of Dentistry, Lebanese University, Beirut, LBN; 2 Faculty of Public Health, Lebanese University, Beirut, LBN

**Keywords:** reactive oxygen species, circadian rhythm, periodontal diseases, antioxidant activity, saliva

## Abstract

Background

Saliva has a powerful antioxidant activity proposing that it might have a protective role in the oral cavity. It is yet unclear, how circadian rhythm might affect this activity.

Objective

The main goal of this study was to compare the antioxidant status of saliva in patients with periodontal diseases (PD) to that of healthy people on a diurnal basis.

Material and methods

A total of 18 periodontal healthy individuals and 18 patients with chronic periodontitis were chosen. Samples of saliva were collected in the morning between 6:00 and 8:00 and in the evening between 6:00 and 8:00 (both stimulated and non-stimulated). The amount of glutathione (GSH), malondialdehyde (MDA), and total antioxidant status (TAS) in the salivary samples were analyzed, and its flow was also assessed. In addition, the scavenging capacity of saliva was tested in three systems generating oxygen free radicals.

Results

Results showed that GSH and TAS concentrations in the evening saliva of healthy subjects were significantly higher than those in the morning saliva, while MDA levels decreased (p<0.05). Conversely, there was no significant increase in GSH and TAS levels in the evening saliva of subjects with PD, and lipid peroxidation remained constant. On the other hand, the evening saliva of healthy subjects but not of subjects with PD was significantly more potent in scavenging free radicals in vitro than the morning saliva, especially for the superoxide (O2.-) radical (p<0.05). Moreover, scavenging activity was higher in stimulated than non-stimulated saliva. This activity was higher in evening saliva compared to the morning one and greater in healthy subjects compared to patients with PD (p<0.05).

Conclusion

A balance exists between oxidative stress and antioxidant mechanisms to maintain homeostasis in the oral cavity. This balance is deregulated in patients with PD as their saliva is unable to properly scavenge free radicals that might potentially increase over the day. Antioxidant supplements may be used in accordance with the circadian rhythm to minimize oxidative damage.

## Introduction

Saliva is a biological fluid essential for maintaining oral health and for the achievement of many oral functions (mastication, taste, digestion, phonation, etc.). It also provides an easily available, noninvasive diagnostic medium for a wide range of diseases and clinical situations [1]. Saliva is the principal defensive factor in the mouth, and a reduction in its flow rate affects oro-dental health [2]. Inflammatory periodontal disease (PD) is among the most common chronic diseases affecting the periodontium that surrounds and supports the teeth. The cause of PD is thought to be an imbalance in the bacterial species that populate the mouth cavity, with the host immunological response to these bacterial pathogens resulting in the destruction of the epithelia and other structures of the periodontal tissues, as well as inactivating repair systems [3].

In 1991, Guarnieri et al. were the first to find a large concentration of polymorphonucleocytes (PMN) cells at the site of gingival inflammation [4]. The authors hypothesized that superoxide radicals (O2.-) generated by PMN during the host immunological response could induce oxidative damage to host tissues depending on the variations of the rate of O2.- generation relative to the inherent antioxidant capacity. Since then, many studies have revealed that the peripheral blood neutrophils of patients with periodontitis consistently show elevated production of reactive oxygen species [5].

In this context, antioxidants are present in all body fluids and tissues in order to protect them against endogenously formed free radicals. Saliva contains both antioxidant enzymes such as glutathione peroxidase, catalase, superoxide dismutase (SOD) and non-enzymatic antioxidants such as uric acid, albumin, and lactoferrin providing protection within cells; they are also present along with vitamins in extracellular fluids, including saliva [6]. PD has been diagnosed primarily through clinical and radiographic measurements of periodontal tissue loss. These metrics may measure past degradation and are of limited use in early diagnosis [7]. However, total antioxidant activity or status (TAS) in the saliva of PD patients has been found to be lower than that of non-PD individuals associated with increased oxidative stress [8,9]. Likewise, oxidative stress has been reported to increase with periodontitis [10]. PD is clearly an important and potentially life-threatening condition that is often underestimated by health professionals and the general population. The available evidence implicating inflammatory mediators and cells in the disease process suggests that local antioxidant status may be of importance in determining susceptibility to the disease and its progression following initial bacterial colonization [11].

Despite the valuable work done in this area mainly by Sculley and Langley-Evans [12], there is still a facet to explore, namely the diurnal variation of the antioxidant activity of saliva. Currently, biorhythms occupy an important place in research. In fact, saliva secretion follows circadian rhythms, as well as respiratory and motor activities, which directly determine the production of free radicals. These activities are diurnally variable, like the rate of lipid peroxidation, the activities of some antioxidant enzymes, and the amounts of low-molecular-weight antioxidants [13,14].

This research aims to investigate in vitro the effect of the circadian rhythm on the TAS of stimulated and unstimulated saliva of patients with PD versus individuals without PD in order to fill the gap in this area based on the bibliography above-mentioned.

## Materials and methods

Participants and saliva collection

This study included 36 male participants: 18 patients with chronic periodontitis (aged 40 to 60 years, with a mean age of 52.5) and 18 healthy controls without PD (aged 37 to 59 years, with a mean age of 48.5). The subjects were chosen from individuals who applied to Lebanese University, Faculty of Dentistry, Department of Periodontology, due to periodontal concerns or for routine checks. One examiner was responsible for all the clinical measurements and saliva sampling.

To calculate the minimal sample size, we used the following formula: n= Z2 P(1-P)/d2 (where n is the sample size, Z is the statistic corresponding to the level of confidence, P is the expected prevalence that can be obtained from the same studies or a pilot study conducted by the researchers, and d is precision corresponding to effect size). A number of 16 per group was thus sufficient to show the intended difference, based on our previous work [10].

Clinical data were recorded from all teeth except third molars and severely malpositioned teeth. Chronic periodontitis patients were chosen based on four criteria:

- Probing pocket depth >4 mm recorded from four surfaces of each tooth distobuccal, mid-buccal, mesiobuccal, and mid-lingual using a graduated periodontal probe (Hu Friedy, Chicago, IL, USA). The probe was inserted parallel to the vertical axis of the tooth and rotated circumferentially around each surface of each tooth to detect the areas of deepest penetration. Pocket depth equals the distance from the free gingival margin to the pocket base.

- The bleeding index was calculated as the percent of bleeding points recorded 30 seconds after probing. The bleeding on probing was performed by gentle insertion of the periodontal probe tip into the sulcus, followed by a gentle sweep around from one proximal surface to the opposite proximal surface. The number of sites where bleeding is recorded is divided by the total number of available sites in the mouth and multiplied by 100 to express the bleeding index as a percentage.

- Moderate to severe gingival inflammation through the following symptoms: glazing, redness, edema, hypertrophy of the marginal or papillary gingival unit, congestion, or ulceration.

- Thirty percent of bone loss using dental X-rays. We measured, in millimeters, the distance between the cement-enamel junction and the alveolar bone crest, as well as the distance between the cement-enamel junction and the root apex. Bone loss percentage was determined by the difference between those distances multiplied by 100.

Periodontally healthy subjects were selected as follows: probing pocket depth ≤1.5 mm with no bleeding on probing, no bone loss, and no signs of gingival inflammation (redness, clinical swelling, edema, or pain).

Periodontal healthy patients were chosen since they had no history of PD or gingival inflammation and maintained good oral hygiene [15]. The individuals in the study were nonsmokers, not obese with no sleep disorders, and no history of systemic disease. They had not received periodontal therapy, had not taken antibiotics, anti-inflammatory medicines, or any other drugs for at least six months, and did not consume alcohol or antioxidants (Table 1). Participants were asked to refrain from eating, drinking, or engaging in oral hygiene procedures for at least four hours prior to saliva collection. The Central Research Committee of our university approved this study, and all participants provided informed consent.

**Table 1:** General information on subjects giving saliva samples PD: Patients with periodontal diseases, HS: Healthy subjects

Parameters	PD	HS
Number of subjects	18	18
Age (year)	40-60 (52.5 ± 4.54)	37-59 (48.5 ± 4.39)
Body Mass Index (kg/m^2^)	20-29.4 (25.4± 3.2)	20-30.4 (25.3 ± 3.1)
Smokers	No	No
Chronic systemic disease	No	No
Probing depth (mm)	5.4 ± 0.8	1.28 ± 0.19
Bleeding on probing (%)	41 ± 2.8	0
Periodontal sites involved	4	4

Saliva samples were collected in a quiet room between 6:00 and 8:00 in the morning, and similarly between 6:00 and 8:00 in the evening. Unstimulated whole saliva was collected for a 10-minute time span. As the subject was seated at a low table with the head down and mouth slightly open, saliva was allowed to drip from the lower lip into a small plastic funnel and from there into a graduated 10 ml tube in dry ice. No other conscious movements of the oral musculature were made during the collection. Stimulated saliva was collected in the same way after chewing paraffin gum for 15 minutes. Saliva samples were centrifuged immediately to remove cell debris (1000 x g for 10 minutes at 4°C). The supernatant was removed and stored in small aliquots at -80 °C until analysis.

Saliva analysis

All reagents used were of analytical grade, mostly from Sigma (Sigma-Aldrich, St. Louis, USA) apart from the colorimetric kits.

Determination of Glutathione in Saliva (GSH)

We used a colorimetric assay kit from Oxford Biomedical Research (USA) employing a kinetic enzymatic recycling test based on the oxidation of GSH by 5,5'-dithiobis-(2-nitrobenzoic acid) (DTNB) to measure the total glutathione (tGSH) content of biological samples. According to the manufacturer’s instructions and some modifications described previously [16], GSH standards or saliva samples were placed in the microtiter plate wells, followed by DTNB and GSH reductase. The addition of NADPH2 to the wells initiated the progressive reduction of DTNB by GSH, resulting in a color intensification measured at 405 nm. The pace of color change, which normally occurs during a four-minute period, is proportional to the tGSH concentration. As a result, the content of tGSH in unknown samples can be calculated by referring to the standard curve. GSH interacted with DTNB to form both a colorful ion that absorbed light at 405 nm and a mixed disulfide. The disulfide interacted with more GSH to produce another ion, glutathione disulfide (GSSG). The latter is enzymatically reduced to GSH, which subsequently re-enters the cycle. Because GSSG accounts for a minor percentage of total acid solution-free GSH, the results for tGSH (which includes both GSH and GSSG) are represented in mg/dl.

Total Antioxidant Status of Saliva (TAS)

The TAS was estimated by the 2,2'-azino-bis(3-ethylbenzothiazoline-6-sulfonic acid (ABTS) assay (Randox Lab, Crumlin, UK) as previously described [17]. This method used the ferrylmyoglobin radical, which is formed when metmyoglobin is activated, to interact with phenothiazine. The ABTS radical cation was generated via ABTS (2,2'-azinobis(3-ethylbenzo-thialozine-6-sulphonic acid)). This blue chromogene showed remarkable absorption at 660 nm. In the presence of antioxidants, the production of ABTS radical cations was reduced. As a result, absorption was inhibited in proportion to total antioxidant capacity. The assay was standardized with Trolox, a vitamin E Analog.

Lipid Peroxidation

Malondialdehyde (MDA) levels were measured in the saliva samples by the method of Jain et al. (1989) and as described previously [18,19]. This method relies on the interaction of MDA with thiobarbituric acid (TBA) to form a complex that can be measured using a spectrophotometer. A sample of 0.2 ml was well mixed with 0.8 ml of phosphate-buffered saline (pH 7.4) and 0.025 ml of butylated hydroxytoluene (BHA) solution (0.88%). After adding 0.5 mL of 30% trichloroacetic acid (TCA), the samples were stored on ice for two hours before centrifuging at 2000 x g for 15 minutes at 25 °C. One milliliter of supernatant was combined with 0.075 milliliters of 0.1 M ethylenediaminetetraacetic acid (EDTA) and 0.25 milliliters of 1% TBA in 0.05 N NaOH. The samples were immersed in boiling water for 15 minutes, then cooled to room temperature, and the absorbance was measured at 532 nm.

Scavenging Activity of Saliva In Vitro

Generation of free radicals by electrolysis (ELS): The free radical scavenging capacity of saliva was evaluated in vitro using ELS (two platinum wire electrodes placed in a 20 ml Tyrode buffer solution to generate ROS and their by-products). A constant 10 mA direct current generated by a stimulator was applied for five minutes in the absence or presence of 500 μl of saliva from each sample. The amount of oxygen-free radicals (OFR) and their by-products were determined by a colorimetric method using N, N-diethyl-P-phenylendiamine (DPD). A volume of 1 ml of ELS sample was added to 2 ml of DPD (25 mg/ml) dissolved in the buffer at the end of ELS. The ELS-induced oxidant species reacted instantly with the DPD reagent to produce a red color measurable at 510 nm [20].

Generation of superoxide radical: The superoxide radical scavenging activity was performed according to the method used in our laboratory [8] and described also by Martinez et al. [21] using the xanthine-xanthine oxidase system (XXO). This method is based on the fact that xanthine was converted to uric acid by the enzyme xanthine oxidase, resulting in the formation of the by-product superoxide. Superoxide combined with nitroblue tetrazolium (NBT, 5 mg/ml buffer) formed a formazine blue color measured at 560 nm. The antioxidant present in the saliva samples scavenged the superoxide and the reduction in color was proportional to the antioxidant content in the sample. An amount of 50 μl of xanthine and 20 μl of NBT were added to 500 μl of saliva or standard. The volume was made up to 1 ml with phosphate buffer (50 mM, pH 7.5). An amount of 50 μl of xanthine oxidase was added to the system, mixed well to start the reaction, and incubated at 37° C for 30 minutes in a water bath. The reaction was stopped after 30 minutes by adding 100 μl of 0.1 N HCl. A standard curve was made using 1.0-100 units/ml of bovine SOD. The absorbance of a blank (control) prepared without saliva or a standard was considered to be 100% radical production.

Generation of hydroxyl radical: The deoxyribose assay (DOR) was used to determine the hydroxyl radical scavenging activity in an aqueous medium [22]. The reaction mixture containing FeCl3 (100 μM), EDTA (104 μM), H2O2 (1 mM), and 2-deoxy-D-ribose (2.8 mM) was mixed with or without 500 μl of saliva (10-250 μg) in a 1 ml final reaction volume made with potassium phosphate buffer (20 mM, pH 7.4) and incubated for 1 hour at 37°C. The mixture was heated at 95°C in a water bath for 15 minutes, followed by the addition of 1 ml each of TCA (2.8%) and TBA (0.5% TBA in 0.025 M NaOH) containing 0.02% 2-Deoxy-D-ribose, BHA. Finally, the reaction mixture was cooled on ice and centrifuged at 5000 rpm for 15 min. The absorbance of the supernatant was measured at 532 nm. The negative control without saliva was considered 100 DOR oxidation.

Statistical analysis

Data were presented in mean ± standard deviation and analyzed by analysis of variance (ANOVA), followed by the Student's t-test or Tukey’s test. Statistical analysis used the Statistical Package for the Social Sciences (IBM SPSS Statistics for Windows, IBM Corp., Version 23.0, Armonk, NY). The p-value < 0.05 was statistically significant.

## Results

Saliva flow rate

The flow rate of stimulated saliva is approximately three times higher than the unstimulated (resting) flow in healthy subjects and patients with PD as well. In stimulated saliva, the flow rate of evening saliva is significantly higher than morning saliva in healthy subjects (1.89±0.27 versus 1.65±0.22; p<0.05), but not in patients with PD (1.63±0.21 versus 1.57±0.24). The same pattern is observed in unstimulated saliva, although in a lesser range (Table 2).

**Table 2:** Salivary flow rate (ml/min) of stimulated and unstimulated saliva of healthy subjects (HS) and patients with periodontal diseases (PD) * p<0.05 PD versus HS, # p<0.05 evening versus morning saliva

Saliva	Time	Subjects	Flow rate (ml/min)
Stimulated	Morning	HS	1.65±0.22
		PD	1.57±0.24
	Evening	HS	1.89±0.27#
		PD	1.63±0.21*
Unstimulated	Morning	HS	0.51±0.08
		PD	0.48±0.09
	Evening	HS	0.82±0.13#
		PD	0.55±0.10*

Determination of the antioxidants in saliva

As shown in Table 3, compared to healthy subjects, patients with PD have a significant decrease in GSH (1.125±0.17 versus 0.847±0.08; p<0.05) and TAS (220.34±27.06 versus 137.66±22.57; p<0.05) and increased MDA (1.15±0.48 versus 5.83±0.52) in stimulated saliva collected in the morning. On the other hand, TAS increased and MDA decreased in the evening saliva of healthy subjects compared to the morning saliva, but not in the saliva of patients with PD (p<0.05). It is noteworthy to note that GSH, TAS, and MDA concentrations are 30% to 50% higher in stimulated saliva versus unstimulated saliva, with the same pattern but in a lesser range in unstimulated saliva.

**Table 3:** Determination of glutathione (GSH), lipid peroxidation in terms of malondialdehyde (MDA), and total antioxidant status (TAS) of morning and evening stimulated and unstimulated saliva of healthy subjects (HS) and subjects with periodontal diseases (PD) * p<0.05 PD versus HS, # p<0.05 evening versus morning saliva

Saliva	Time	Subjects	GSH (mg/dl)	MDA (nmol/mL)	TAS (nmol/mL)
Stimulated	Morning	HS	1.125±0.17	4.15±0.48	220.34±27.06
		PD	0.847±0.08*	5.83± 0.52*	137.66±22.57*
	Evening	HS	1.293±0.15#	4.98±0.50#	258.65±19.41#
		PD	0.883±0.09*	7.26±0.68*	141.32±17.65*
Unstimulated	Morning	HS	0.816±0.15	3.18±0.45	165.28±30.23
		PD	0.629±0.07*	4.25±0.48*	125.17±21.18*
	Evening	HS	0.932±0.11#	3.62±0.46	183.27±16.58
		PD	0.679±0.07*	5.27±0.60*	132.74±18.83*

Scavenging activity of saliva

**Figure 1:**
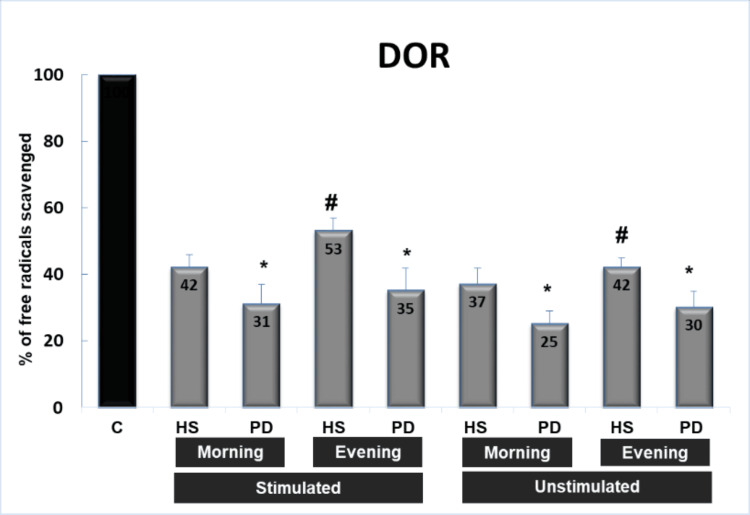
Scavenging effect of saliva (stimulated or unstimulated; collected in the morning or evening) against a burst of free radicals generated by the deoxyribose system (DOR) C: control sample without saliva, HS: healthy subjects, PD: patients with periodontal diseases * p<0.05 PD versus HS, # p<0.05 evening versus morning saliva

**Figure 2:**
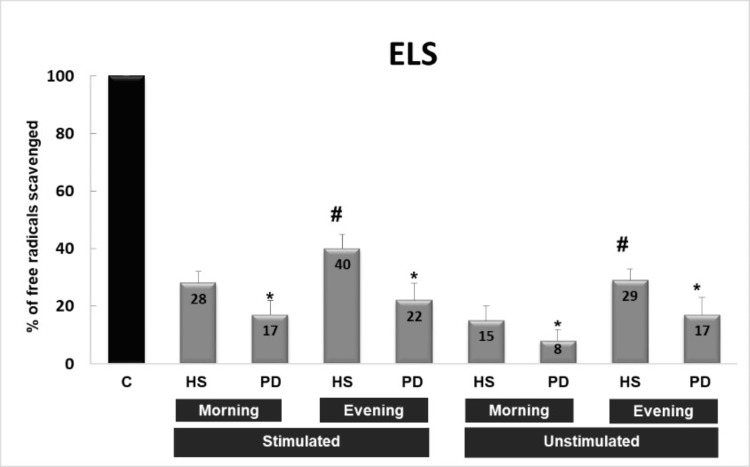
Scavenging effect of saliva (stimulated or unstimulated; collected in the morning or evening) against a burst of free radicals generated by electrolysis (ELS) C: control sample without saliva, HS: healthy subjects, PD: patients with periodontal diseases * p<0.05 PD versus HS, # p<0.05 evening versus morning saliva

**Figure 3:**
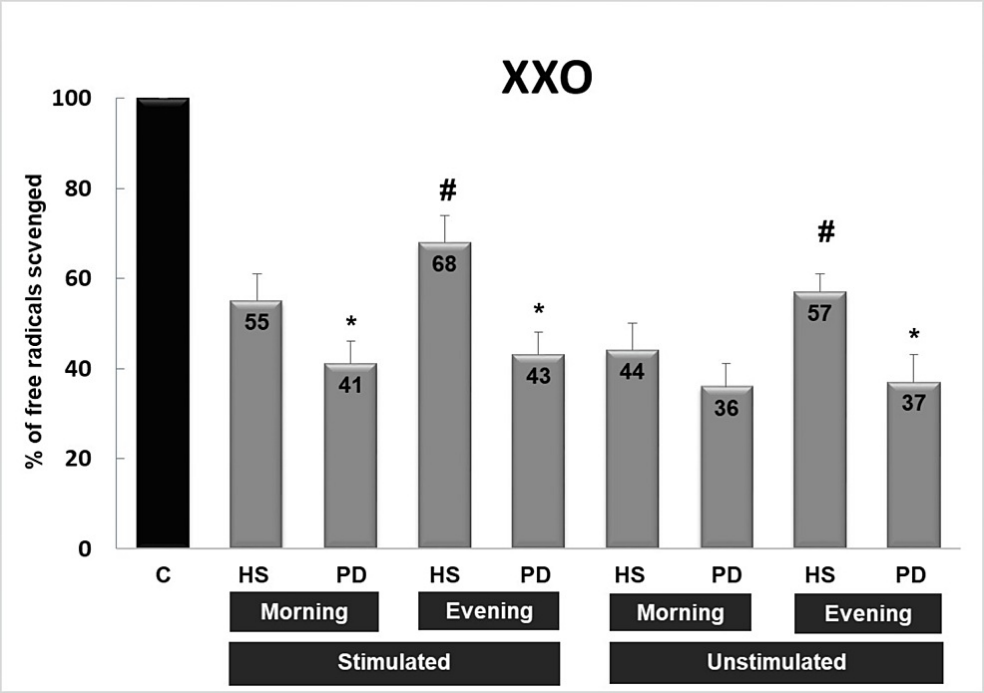
Scavenging effect of saliva (stimulated or unstimulated; collected in the morning or evening) against a burst of free radicals generated by xanthine/xanthine oxidase system (XXO) C: control sample without saliva, HS: healthy subjects, PD: patients with periodontal diseases * p<0.05 PD versus HS, # p<0.05 evening versus morning saliva

Three systems were used to generate free radicals (Figures 1-3): ELS, DOR, and XXO. Stimulated and unstimulated saliva samples were collected in the morning and evening from healthy subjects and PD patients. In the three systems, saliva is able to significantly inhibit the absorbance (reflecting the scavenging capacity of saliva against free radicals) from 8% to 68% in comparison with control values (100% free radical generation). On the other hand, the capacity of stimulated saliva to scavenge free radicals in the three systems is higher than that of unstimulated saliva, and both followed the same pattern. Moreover, the saliva of healthy subjects possesses a higher capacity to scavenge free radicals, mainly against superoxide radicals, than the saliva of patients with PD (p<0.05). The most important finding is that the scavenging capacity of evening saliva in healthy subjects is significantly higher than that of morning saliva (p<0.05), while in patients with PD, evening and morning saliva possess approximately the same capacity to scavenge OFR.

## Discussion

To date, research on salivary antioxidant levels in PD has produced inconsistent results. This may be a consequence of the different methods used by researchers, but there are additional aspects that could explain the differences [23]. As previously indicated, the reference work done in this field is that of Guarnieri et al. [4], who observed increased concentrations of PMN at sites of gingival inflammation, if they were not matched by an increase in antioxidant concentration. Moore et al. [24] then used the Trolox Equivalent Antioxidant Capacity assay to measure the antioxidant capacity of both stimulated and unstimulated saliva in healthy individuals and PD patients, and there was no change in saliva antioxidant capacity. However, PD patients' sample size was limited (seven subjects), and their disease state was unclear, as they were simply reported as requiring dental treatment. Furthermore, saliva samples were held at -20°C, which may have resulted in antioxidant breakdown.

In 1997, Chapple et al. [25] conducted a study on serum and saliva samples from patients and healthy individuals. The two groups were clearly identified, with 18 periodontal cases and 16 controls. The serum antioxidant capacity of both groups was found to be identical. However, salivary antioxidant capacity was significantly lower in PD patients than in controls. This would suggest that the decrease in salivary antioxidant activity is either due to an increase in free radical concentration (where scavenging antioxidants are absorbed in the quench of a free radical chain reaction), or that the increased free radical activity is due to lower salivary antioxidant levels.

In 2003, Sculley and Langley-Evans [12] as well as Diab-Ladki et al. [8] from our laboratory demonstrated that PD was associated with lower antioxidant capacity in saliva. On the other hand, Guentsch et al. [26] showed that patients with periodontitis exhibit more lipid peroxidation than healthy subjects, and that effect was enhanced by smoking. More recently, Yoshino et al. [27], using Electron Spin Resonance, demonstrated that evaluation of the salivary antioxidant activity towards O.2 might be an effective parameter for the objective assessment of PD progression. Therefore, an imbalance between oxidative stress and antioxidant capacity played a crucial role in the pathogenesis of PD [28,29], but it still needed to be explored.

Based on these previous works, we have elaborated on an original study related to the antioxidant activity of saliva in patients with PD compared to healthy subjects. Two important factors were taken into account and linked together: (1) the chronobiological aspect of the antioxidant activity of saliva and (2) the salivary flow by collecting both stimulated and unstimulated samples. Particular attention has been given to each detail in choosing the patients with PD as well as the healthy subjects. In fact, inclusion criteria were very severe in terms of age, weight, medical history, meals, and psychological stress; every subject who did not fit those criteria was discarded from the study. Since salivary flow was crucial for our results, samples were taken after a period of rest during one week of June 2019, at the same time and in the same place; they were selected under the control of the same specialist who had given an explanation and demonstration to ensure the conformity of our samples. Meanwhile, all biochemical tests were done by the same technician in triplicate.

When the mouth is in a resting state, without the stimulation of the exogenous glands (chewing) that is associated with mechanical stimulation, the flow of saliva is slow, which helps to moisten the mouth and lubricate the mucous membranes. Unstimulated saliva plays an important role in maintaining the oral cavity’s health and well-being. It also plays a significant role in protecting the oral cavity from dental caries (dental caries). When the flow rate is higher, the clearance is faster. When the buffer capacity is higher, there are fewer microbial attacks. It is important to note that unstimulated saliva contains fewer antioxidants than stimulated saliva; however, when the flow rates are considered, the antioxidant capacity is greater than that of unstimulated saliva. This allows for a more accurate measurement of the delivery of antioxidants to the oral region using saliva as a vector, as opposed to taking a single sample. The environment inside the mouth is not static, it is subject to continuous variations [6].

Concerning diurnal rhythm, this is an important factor that must be taken into consideration. In fact, we possess a circadian clock, an endogenous timing system that generates biochemical, physiological, and behavioral rhythms. Some studies were conducted many years ago to shed light on the diurnal variation of salivary composition and secretion as well as the variation of its antioxidant activity [13,14]. In fact, research in this area remained timid, and no particular study was devoted to exploring this rhythmic activity in patients with PD. However, recently, research in this domain has been reinitiated [30].

In order to strengthen our hypothesis, we measured GSH concentration, an important redox regulator in saliva, whose maintenance is essential for periodontal health. Lipid peroxidation is the process of oxidative lipid damage that participates in the development of various diseases, including periodontitis, and MDA is its terminal product. The total antioxidant capacity is a biomarker often used in serum and saliva in order to investigate oxidative stress in many pathological conditions. On the other hand, we generate free radicals via three systems: ELS which generates a burst of different radicals, XXO which generates mainly superoxide radicals, and DOR which generates hydroxyl radicals.

Oxidative stress has been reported to follow a circadian rhythm, therefore the production of antioxidants and protective enzymes should be regulated or expressed in rhythmic fashions. As a matter of fact, biorhythmic clock disruption can destroy host defense responses and deregulation of the immune system, which may be a pathogenesis mechanism that leads to various diseases. In support of our results, it has been shown recently in rats that brain and muscle aryl hydrocarbon receptor nuclear translocator-like-1(BMAL1), a core component of the circadian clock is regulating intracellular redox status and is associated with the progression of periodontitis thereby exacerbating oxidative stress and apoptosis in periodontal tissues [30].

In our adopted conditions, the results obtained are comparable with those reported by most researchers in the literature concerning the decrease of antioxidant activity in patients with PD versus healthy patients. However, the main finding of our study is that the antioxidant activity of healthy subjects increased significantly in the evening saliva compared to the morning saliva, whereas this activity did not increase significantly in patients with PD. This is also the same for stimulated versus resting saliva, but the antioxidant capacity of the latter is less effective. Superoxide radicals seem to be scavenged more easily by saliva than hydroxyl radicals. Therefore, it is clear that individuals suffering from PD were unable to enhance their salivary antioxidant activity in order to scavenge free radicals, which could possibly be created in greater quantities during the day.

## Conclusions

Antioxidant activity of healthy subjects increased in afternoon saliva versus morning saliva probably to fight the oxidative process increasing in the oral cavity during the day while antioxidant activity of saliva remained unchanged in PD patients suggesting the significance of rhythmicity in avoiding excessive oxidative stress. There is a delicate balance between the naturally occurring pro-oxidants and antioxidants in the oral cavity that are deregulated in PD patients. This balance is expected to provide novel therapeutic approaches to raise oral hygiene awareness and thus help to identify new treatment options to lower the risk of comorbidities associated with periodontitis. As an outlook for the future, to boost the antioxidant capacity of the saliva, antioxidant supplementation using a sugar-free chewable gum may be used as a time-based therapy or preventative treatment for these diseases in the oral cavity.

## References

[1] Zhang CZ, Cheng XQ, Li JY, Zhang P, Yi P, Xu X, Zhou XD (2016). Saliva in the diagnosis of diseases. Int J Oral Sci.

[2] Pedersen AM, Sørensen CE, Proctor GB, Carpenter GH, Ekström J (2018). Salivary secretion in health and disease. J Oral Rehabil.

[3] Beck JD, Papapanou PN, Philips KH, Offenbacher S (2019). Periodontal medicine: 100 years of progress. J Dent Res.

[4] Guarnieri C, Zucchelli G, Bernardi F, Scheda M, Valentini AF, Calandriello M (1991). Enhanced superoxide production with no change of the antioxidant activity in gingival fluid of patients with chronic adult periodontitis. Free Radic Res Commun.

[5] Magán-Fernández A, Rasheed Al-Bakri SM, O'Valle F, Benavides-Reyes C, Abadía-Molina F, Mesa F (2020). Neutrophil extracellular traps in periodontitis. Cells.

[6] Toczewska J, Konopka T (2019). Activity of enzymatic antioxidants in periodontitis: a systematic overview of the literature. Dent Med Probl.

[7] Wang J, Schipper HM, Velly AM, Mohit S, Gornitsky M (2015). Salivary biomarkers of oxidative stress: a critical review. Free Radic Biol Med.

[8] Diab-Ladki R, Pellat B, Chahine R (2003). Decrease in the total antioxidant activity of saliva in patients with periodontal diseases. Clin Oral Investig.

[9] Toczewska J, Maciejczyk M, Konopka T, Zalewska A (2020). Total oxidant and antioxidant capacity of gingival crevicular fluid and saliva in patients with periodontitis: Review and clinical study. Antioxidants (Basel).

[10] Chen M, Cai W, Zhao S (2019). Oxidative stress-related biomarkers in saliva and gingival crevicular fluid associated with chronic periodontitis: a systematic review and meta-analysis. J Clin Periodontol.

[11] Zhang T, Andrukhov O, Haririan H, Müller-Kern M, Liu S, Liu Z, Rausch-Fan X (2015). Total antioxidant capacity and total oxidant status in saliva of periodontitis patients in relation to bacterial load. Front Cell Infect Microbiol.

[12] Sculley DV, Langley-Evans SC (2003). Periodontal disease is associated with lower antioxidant capacity in whole saliva and evidence of increased protein oxidation. Clin Sci (Lond).

[13] Hardeland R, Coto-Montes A, Poeggeler B (2003). Circadian rhythms, oxidative stress, and antioxidative defense mechanisms. Chronobiol Int.

[14] Wilking M, Ndiaye M, Mukhtar H, Ahmad N (2013). Circadian rhythm connections to oxidative stress: implications for human health. Antioxid Redox Signal.

[15] Caton JG, Armitage G, Berglundh T (2018). A new classification scheme for periodontal and peri-implant diseases and conditions - introduction and key changes from the 1999 classification. J Clin Periodontol.

[16] Öngöz Dede F, Bozkurt Doğan Ş, Balli U, Avci B, Durmuşlar MC, Baratzade T (2016). Glutathione levels in plasma, saliva and gingival crevicular fluid after periodontal therapy in obese and normal weight individuals. J Periodontal Res.

[17] Chahine N, Nader M, Chalhoub W, Chahine R (2020). Natural antioxidants and vitamins supplementation shelters adolescents from upper respiratory tract infection. Int J Child Health Nutr.

[18] Jain SK, McVie R, Duett J, Herbst JJ (1989). Erythrocyte membrane lipid peroxidation and glycosylated hemoglobin in diabetes. Diabetes.

[19] Chahine N, Hanna J, Makhlouf H, Duca L, Martiny L, Chahine R (2013). Protective effect of saffron extract against doxorubicin cardiotoxicity in isolated rabbit heart. Pharm Biol.

[20] Lecour S, Baouali AB, Maupoil V (1998). Demonstration of the production of oxygen free radicals during electrolysis. Free Radicals Biol Med.

[21] Martinez CA, Loureiro ME, Oliva MA, Maestri M (2001). Differential responses of superoxide dismutase in freezing resistant Solanum curtibolum and freezing sensitive Solanum tuberosum subjected to oxidative and water stress. Plant Sci.

[22] Halliwell B, Gutteridge JM, Aruoma OI (1987). The deoxyribose method: a simple test-tube assay for determination of rate constants for reactions of hydroxyl radicals. Anal Biochem.

[23] Sczepanik FS, Grossi ML, Casati M, Goldberg M, Glogauer M, Fine N, Tenenbaum HC (2020). Periodontitis is an inflammatory disease of oxidative stress: we should treat it that way. Periodontol 2000.

[24] Moore S, Calder KA, Miller NJ, Rice-Evans CA (1994). Antioxidant activity of saliva and periodontal disease. Free Radic Res.

[25] Chapple IL, Mason GI, Garner I, Matthews JB, Thorpe GH, Maxwell SR, Whitehead TP (1997). Enhanced chemiluminescent assay for measuring the total antioxidant capacity of serum, saliva and crevicular fluid. Ann Clin Biochem.

[26] Guentsch A, Preshaw PM, Bremer-Streck S, Klinger G, Glockmann E, Sigusch BW (2008). Lipid peroxidation and antioxidant activity in saliva of periodontitis patients: effect of smoking and periodontal treatment. Clin Oral Investig.

[27] Yoshino F, Yoshida A, Wada-Takahashi S (2012). Assessments of salivary antioxidant activity using electron spin resonance spectroscopy. Arch Oral Biol.

[28] Shang J, Liu H, Zheng Y, Zhang Z (2023). Role of oxidative stress in the relationship between periodontitis and systemic diseases. Front Physiol.

[29] Edgar WM (1992). Saliva: its secretion, composition and functions. Br Dent J.

[30] Liu X, Cao N, Liu X (2022). Circadian rhythm disorders aggravate periodontitis by modulating BMAL1. Int J Mol Sci.

